# Unraveling motives: identifying the impact of university attendance motives on learning behaviors among dental students

**DOI:** 10.1186/s40359-024-01846-y

**Published:** 2024-06-14

**Authors:** Yongmin Shin, Jaehee Rho, Minhae Cho, Minjung Lee, Ye Ji Kang, Jungjoon Ihm

**Affiliations:** 1 Dental Research Institute, School of Dentistry, Seoul National University, Gwanak-ro 1, Gwanak-gu, Seoul, Republic of Korea; 2 Department of Education, College of Educational Science, Yonsei University, Seoul, Korea; 3 School of Social Work, University of Memphis, Memphis, USA; 4 Yale School of Nursing, Yale University, New Haven, CT USA; 5 Department of Medical Education, Hanyang University College of Medicine, Seoul, Korea; 6 Department of Dental Education, Seoul National University, Seoul, South Korea

**Keywords:** University attendance motives, Learning behavior, Dental students, Academic engagement, Deep approach to learning, Growth mindset

## Abstract

**Purpose:**

Students report various motives for attending university (MAU) grouped under five categories, namely, personal–intellectual development (PER), humanitarian (HUM), careerist–materialist (CAR), expectation-driven (EXP), and uncertain motives. Although the literature demonstrates that these motives exert an influence on learning and achievement, relatively less attention is given to this issue in the context of dental students. This study aimed to examine the relationship among the mindsets, MAU, academic engagement (AE), and DAL of dental students and to test the mediating effect of AE on the relationship between MAU and deep approach to learning (DAL).

**Methods:**

The study recruited 226 dental students at various levels of the curriculum, who responded to four questionnaires for measuring MAU, DAL, mindsets, and AE. The study employed structural equation modeling to analyze the mediation effects of AE on the relationship between MAU and DAL and to determine the influence of mindsets on MAU.

**Results:**

This model reveals the significant relationships of a growth mindset with CAR, PER, and HUM. Moreover, the study finds that a fixed mindset was associated with CAR, EXP, and uncertain motives. Furthermore, AE only fully mediated the significant positive relationship between PER and DAL, whereas CAR negatively predicted DAL without a mediator.

**Conclusions:**

These findings suggest that administering the inventories in a dental school setting can facilitate a more comprehensive understanding of students’ mindsets toward learning and effective processes related to learning. This understanding can inform instructors’ pedagogical practices, enabling them to provide more effective guidance to students navigating the complexities of academic coursework.

## Introduction

Healthcare students pursue university education for various reasons, including job prospects, income, caring for others, intellectual curiosity, and family expectations [1–4]. Recognizing the multifaceted nature of student motives is crucial, as it serves as a foundation for understanding the complexity that underpins students’ approaches to learning. These insights can be instrumental in developing targeted strategies to enhance student engagement and achievement within the rigorous healthcare education landscape. Despite the importance of understanding the motives of healthcare students, the field of dental education has rarely explored the interrelationship between student motivation to attend dental school and the learning strategies these students employ, as well as their attitudes toward learning. Therefore, this study aims to explore the relationship between the mindsets, MAU, AE, and DAL of dental students and to determine the extent to which AE mediates the relationship between MAU and DAL.

The reasons students enroll in university are closely related to personal aspirations and future goals, including intrinsic (e.g., personal growth) and extrinsic (e.g., social status) desires [5]. Côté and Levine [6] identified five student motives for attending university (MAU): personal–intellectual development (PER; interest in personal growth and intellectual development), humanitarian (HUM; helping others and improving the world), careerist–materialist (CAR; seeking a good career and high socioeconomic status), expectation-driven (EXP; meeting the expectations of family and friends), and default (DEF; attending without a clear reason).

Specifically, PER, HUM, and CAR exhibit positive correlations with a strong commitment to specific goals driven by personal values, whereas EXP and DEF are associated with a state of instability characterized by an inability to fully commit to goals and a continuous search for alternative options [7]. These distinct characteristics may exert varying impacts on educational outcomes. For instance, Côté and Levine [6] found that PER and CAR in the first year predicted self-management and self-motivation in the third year. Additionally, PER and HUM in the first year predicted academic achievement in the third year, while DEF in the first year negatively influenced self-motivation and academic achievement two years later. Based on previous findings, the current study expects that each motive exerts a different impact on learning behaviors, but the link between the motives of dental students and their learning behaviors remains unproven. To address this research gap, the current study explores the unique effects of the MAU of dental students on learning behaviors. Additionally, this study delves into students’ mental frames with specific motives, providing essential evidence for effective interventions to improve academic outcomes.

Mindset is a key motivational factor [8, 9] and influences the manner in which individuals interpret and respond to information [10]. Different mindsets exist in areas such as intelligence [11], personality [12], stress [10], and emotion [13]. This study centers on the intelligence mindset, which offers two contrasting views on its malleability. Students with a growth mindset believe in enhancing intelligence through sustained effort, while those with a fixed mindset consider it unalterable [11]. Therefore, those with a growth mindset exert effort to master challenges with the objective of improving their competence, while individuals with a fixed mindset negatively perceive the value or utility of capacity-enhancing effort and believe in unchanging intellectual abilities, which urges them to pursue performance goals to demonstrate their competence [14]. Prior studies with adolescents have consistently identified a growth mindset as a predictor of positive outcomes, including resilience, school engagement, and cognitive abilities [15–17]. Conversely, a fixed mindset has been linked to negative outcomes such as poor mental health and low grades [18, 19]. 

This study posits that mindset is interrelated with MAU among students. Individuals with a growth mindset focus on personal development. Emphasizing self-growth along with persistent and continuous effort fosters an in-depth understanding of oneself, which facilitates the realization of one’s values and goals and creates a sense of meaning [20–22]. Conversely, individuals with a fixed mindset view mistakes and failure as inherent flaws and avoid challenges. Moreover, they value external achievements, such as GPA or material possessions, over personal growth to validate their self-worth [23–25], which potentially hinders personal value and meaning. Therefore, the study predicts that a growth mindset will positively correlate with PER and HUM (reflecting intrinsic value-based goal commitment), while a fixed mindset will exhibit a positive relationship with CAR, EXP, and DEF (denoting extrinsic value-based goal commitment and lack of self-value in academics).

Healthcare professionals, such as dentists, receive specialized and rigorous education to attain the competency needed for practice in the public domain. Among these students, learning can be categorized into three major approaches: deep approach to learning (DAL), surface approach to learning (SFAL), and strategic approach to learning (STAL). DAL is intrinsically motivated and involves strategies for understanding, seeking meaning, and integrating content and personal experiences, making it an ideal approach. In contrast, SFAL relies on extrinsic motivation and features rote memorization and the reproduction of facts without comprehension [26]. STAL involves adjusting learning behaviors and study habits to align with the course’s assessment demands and instructional format, aiming for high grades while considering the assessment and instructional nature [27]. However, this approach could result in a fragmented understanding of topics and diminished integration of knowledge compared to DAL [28]. Therefore, this study aims to focus on DAL as an exemplary learning behavior based on motivation among dental students.

Differences in attitudes and behaviors toward learning are dependent on the types of personal goals. Research demonstrates that students with intrinsic goals (e.g., intellectual growth and helping others) engage in DAL more and perform better than those with extrinsic goals (e.g., financial gain and reputation) [29–33]. Based on these findings, the study predicts that students with PER and HUM are more likely to adopt DAL compared with the other motives. However, noting the existence of a psychological mechanism that connects the motives of students and DAL is crucial. Drawing on the Presage–Process–Product model by Biggs [26, 29], engagement in the learning process is underscored as a fundamental prerequisite for DAL. Engagement is inherently linked to psychological and motivational states; thus, one can reasonably infer that this construct operates as a mediator between MAU and DAL.

The literature provides strong theoretical and empirical evidence that intrinsic motivation is a critical factor in fostering or predicting academic engagement (AE), which is composed of three components, namely, vigor, dedication, and absorption [34]. Vigor refers to high levels of willingness to exert considerable effort in one’s schoolwork, while dedication indicates a sense of significance and enthusiasm with academic tasks. Finally, absorption is characterized by high levels of concentration and engrossment in educational activities. Previous research demonstrates AE as a strong predictor of positive educational outcomes, including DAL [35], academic adjustment [36], and GPA [37]. In addition, prior studies illustrate that students with intrinsic goals tend to be intrinsically motivated in learning, while those with extrinsic goals tend to be extrinsically motivated [38, 39]. Intrinsically motivated students engage more authentically, while extrinsically motivated ones engage ritualistically [40]. Thus, the study infers that PER and HUM may be positively related to AE, which leads to DAL. Conversely, CAR, EXP, and DEF may have negative or no relationship with AE, which leads to a reduced or no impact on DAL.

The major goal of this investigation is to explore the relationships among the mindsets, MAU, AE, and DAL in dental students and to identify the mediating effect of AE on relationship between MAU and DAL. Toward this end, we aim to determine the effect of mindsets on each motive and which motives significantly predict DAL among dental students through AE, using structural equation modeling (SEM). This examination focuses on motives for selecting their major regardless of academic year instead of motives for university entrance. This approach aims to reveal the reasons and goals underlying their current major choices.

## Materials and methods

### Participants and procedure

The Ethics Committee School of Dentistry at Seoul National University affiliated with the authors (approval No. S-D20210016) reviewed and approved the study. The study used convenience sampling to recruit 298 dental school students in South Korea who provided informed consent. The purpose of this study was introduced to the dental students through a community website in which they are enrolled, as well as during class time, while emphasizing the assurance of confidentiality and anonymity for their participation in this research. From April to May 2021, the participants were invited via email, which included a URL link to a web-based survey (Google Forms), to complete the survey on growth mindset, MAU, AE, and DAL. Prior to commencing the survey, participants were once again apprised of the study’s purpose, the confidentiality of their responses, and the assurance of their anonymity through a notice displayed on the homepage of the web-based survey. The survey had no time limit and lasted for approximately 15–20 min. Out of 298 students, 226 (male: 110; female: 116; *M*_age_ = 22.46; *SD*_age_ = 3.12) completed all measures, and their data were used for analysis. In the pre-clinical course (*n* = 80), 42 1st-year students, 27 2nd-year students, 10 3rd-year students, and 1 4th-year student responded. In the clinical course (*n* = 146), 84 1st-year students and 62 2nd-year students responded. Those who completed the web-based survey in this study were awarded a gift certificate worth $5 as compensation for their participation in the study.

### Measures

#### Growth mindset

The study employed the Korean adapted version of the Theories of Intelligence Scale [41], which was originally developed by Dweck [42], to measure beliefs about growth in intellectual ability through effort and learning. The inventory consists of four items each for growth (e.g., “If you work hard, you can significantly change your ability level.”) and fixed (e.g., “You have a certain amount of ability, and you can’t really do much to change it.”) mindsets, which were rated using a five-point Likert-type scale ranging from 1 = *strongly disagree* to 5 = *strongly agree*. In terms of reliability, Cronbach’s α for the subscales of growth and fixed mindsets were.89 and 0.91, respectively. On the original scale, lower scores indicate a greater prevalence of characteristics associated with a fixed mindset, while higher scores indicate a greater prevalence of characteristics associated with a growth mindset. To date, few studies have identified a specific score cutoff that can be used to distinguish between growth and fixed mindsets. Consequently, the study conceptualizes the growth and fixed mindsets as two separate constructs instead of the opposite poles of a single construct. In this manner, we can separately examine the effect of each mindset on MAU.

#### Motives for attending dental school

The study used the Korean version of Student Motivation for Attending University (SMAU) scale [6, 43] to measure motivation for attending dental school. It modified a few items from the original SMAU scale by changing the word “university” into “major” to evaluate the reasons of the students for selecting their major instead of the university. The SMAU scale consists of 23 items rated using a five-point Likert-type scale ranging from 1 = *strongly disagree* to 5 = *strongly agree* to evaluate five types of motives, namely, CAR (five items, e.g., “My major is a practical means for me to achieve personal success.”), PER (five items, e.g., “My major is satisfying because it gives me the opportunity to study and learn.”), HUM (four items, e.g., “My education should enable me to help people who are less fortunate.”), EXP (five items, e.g., “I am in my major primarily because I am expected to get a degree.”), and DEF (four items, e.g., “I often ask myself why I’m in my major.”). All items were modified to pertain to dental students. The scales reached Cronbach’s α values of 0.86 (CAR), 0.80 (PER), 0.82 (HUM), 0.73 (EXP), and 0.78 (DEF).

### Approaches to learning

The study employed the Korean version of Revised Two-Factor Study Process Questionnaire (R-SPQ-2 F) [44, 45] to assess the level of DAL. Toward this end, the study selected two deep learning approach subscales of R-SPQ-2 F, namely, deep motive for measuring intrinsic interest (e.g., “I find that at times studying gives me a feeling of deep personal satisfaction”) and deep strategy for measuring the maximization of meaning and time management (e.g., “I find most new topics interesting and often spend extra time trying to obtain more information about them.”). These two subscales consist of five items each, which were scored using a five-point Likert-type scale ranging from 1 = *strongly disagree* to 5 = *strongly agree*. In terms of reliability, Cronbach’s α values for DM and DS were 0.68 and 0.73, respectively.

### Academic engagement

To assess AE, the study used the Korean Academic Engagement Inventory (KAEI; Lee and Lee [46]), which was developed and validated on the basis of the engagement concept of Schaufeli et al. [34] The KAEI consists of 16 items rated using a five-point Likert-type scale ranging from 1 = *strongly disagree* to 5 = *strongly agree* and included four subscales, namely, dedication (four items, e.g., “I feel proud when I study.”), vigor (four items, e.g., “I get energy when I study.”), efficacy (four items, e.g., “I have confidence in my studies.”), and absorption (four items, e.g., “Time flies when I study.”). The subscales for dedication, vigor, and absorption are the same as the subcomponents of AE by Schaufeli et al. [34]. However, efficacy is a newly added subscale in the KAEI, which reflects the degree to which people perceive themselves as good at studying. In terms of reliability, Cronbach’s α values reached 0.85 for dedication, 0.87 for vigor, 0.84 for efficacy, and 0.78 for absorption.

#### Model evaluation and estimation

The study applied confirmatory factor analysis (CFA) to confirm the designated factor structure of each measure. In addition to confirming the factor structure using psychometric properties, the study used structural equation modeling (SEM) to examine the associations among the factors of mindsets, MAU, AE, and DAL. In general, SEM consists of two models.

For CFA and SEM, analysis employed the estimates of maximum likelihood with robust standard errors (MLR) using the MLR option in Mplus 8 [47]. The study then analyzed the hypothesized models using approximate fit indices such as root mean square error of approximation (RMSEA), comparative fit index (CFI), and standardized root mean square residuals (SRMR). The study performed descriptive statistics and Pearson’s correlation analysis in Mplus and SPSS to describe the characteristics and relationships of all variables.

## Results

Table 1 presents the descriptive statistics of main factors. In particular, the study examined three statistical indicators, namely, multivariate normality with kurtosis and skewness, multivariate outlier with Cook’s distance, and multicollinearity with variance inflation factors (VIFs). The values for kurtosis and skewness ranged from − 3 to 3, except for one item (3.022), which was not considered problematic. All VIF values were less than 7, which indicates multivariate normality without multicollinearity violations. In addition, all of Cook’s distances were less than 0.095, which is less than the criterion of 1.00 and denotes that multivariate outliers existed in the dataset.

**Table 1:** Descriptive statistics of main factors

Factor	Variable	Mean	SD	Factor	Variable	Mean	SD
Growth mindset	mindset_g1	3.956	0.918	CAR	CAR_1	4.035	0.795
	mindset_g2	3.832	1.043		CAR_2	4.257	0.545
	mindset_g3	4.035	0.804		CAR_3	3.973	0.893
	mindset_g4	4.084	0.617		CAR_4	3.907	0.828
Fixed mindset	mindset_f1	2.226	1.015	PER	PER_1	4.066	0.805
	mindset_f2	2.199	0.947		PER_2	4.027	0.911
	mindset_f3	2.146	0.957		PER_3	4.133	0.832
	mindset_f4	2.248	1.124		PER_4	3.531	1.072
AE	AE_dedi	15.496	10.179	HUM	HUM_1	3.854	0.930
	AE_vig	10.704	14.058		HUM_2	4.124	0.719
	AE_eff	12.429	11.148		HUM_3	4.066	0.850
	AE_abs	10.257	5.748		HUM_4	3.752	1.080
DAL	DAL_1	3.504	0.878	EXP	EXP_1	1.752	0.983
	DAL_2	3.788	1.017		EXP_2	2.062	1.501
	DAL_3	3.518	1.011		EXP_3	1.872	1.165
	DAL_4	2.721	0.838	DEF	DEF_1	1.978	1.252
	DAL_5	3.336	0.869		DEF_2	1.686	0.676
	DAL_6	3.566	0.945		DEF_3	1.823	1.208
					DEF_4	2.336	1.568

*Note* mindset_g: growth mindset, mindset_f: fixed mindset, AE_dedi: dedication as a subscale of Academic Engagement, AE_vig: vigor as a subscale of Academic Engagement, AE_eff: efficacy as a subscale of Academic Engagement, AE_abs: absorption as a subscale of Academic Engagement, CAR: careerist–materialist motive, PER: personal–intellectual development motive, HUM: humanitarian motive, EXP: expectation-driven motive, DEF: default motive, DAL: deep approach to learning

### Confirmatory factor analysis

The overall model fit measured using RMSEA (0.047 < 0.06), CFI (0.923 > 0.90), and SRMR (0.057 < 0.080) indicates *good* (or *acceptable*) fit, which denotes that the factor model fit the data well. The final model exceeded the requirements of at least three indicators per factor. Table 2 depicts that factor correlations ranged from − 0.635 to 0.851, which pertains to discriminant validities among factors.

**Table 2:** Factor correlations among mindset, motivation, AE, and DAL

Factor	Fixed mindset	CAR	PER	HUM	EXP	DEF	AE	DAL
**Growth mindset**	−0.635***	0.378***	0.604***	0.540***	−0.301**	−0.339***	0.712***	0.623***
**Fixed mindset**		−0.063	−0.384***	−0.388***	0.406***	0.531***	−0.385***	−0.339***
**CAR**			0.558***	0.423***	−0.136	−0.209*	0.502***	0.328**
**PER**				0.719***	−0.236*	−0.591***	0.732***	0.630***
**HUM**					−0.274**	−0.437***	0.541***	0.605***
**EXP**						0.765***	−0.266*	−0.334***
**DEF**							−0.471***	−0.467***
**AE**								0.851***

*Note* CAR: careerist–materialist motive, PER: personal–intellectual development motive, HUM: humanitarian motive, EXP: expectation-driven motive, DEF: default motive, AE: academic engagement, DAL: deep approach to learning

*p < .05, **p < .01, ***p < .001

### Structural equation modeling

Based on the confirmed factor structure, the study simultaneously examined the effect of each factor. The overall model fit was within the good or acceptable range (RMSEA = 0.050; CFI = 0.911; SRMR = 0.069). The effects of the growth mindset on the three factors of MAU were significant (b^CAR^ = 0.626, *p* = .000; b^PER^ = 0.677, *p* = .000; b^HUM^ = 0.514, *p* = .000).

**Fig. 1:**
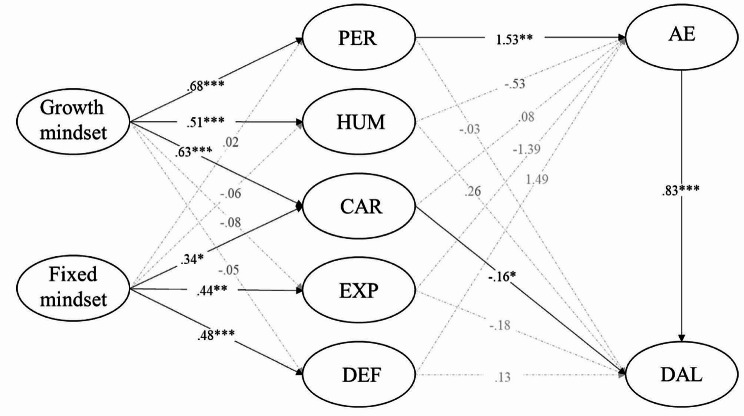
Structural relations of a pathway from mindsets to the DAL. *Note* CAR: careerist–materialist motive, PER: personal–intellectual development motive, HUM: humanitarian motive, EXP: expectation-driven motive, DEF: default motive, AE: academic engagement, DAL: deep approach to learning. Statistically significant paths are represented by continuous lines; broken lines are statistically nonsignificant (*p* > .05). *p < .05, **p < .01, ***p < .001

Figure 1 depicts the filled model with parameter estimates (Table 3), which depicts that the effects of the fixed mindset on the three factors of MAU were significant (b^CAR^ = 0.336, *p* = .017; b^EXP^ = 0.441, *p* = .001; b^DEF^ = 0.476, *p* = .000). Moreover, the effects of CAR (b^DAL^ = − 0.164, *p* = .048) and PER (b^engagement^ = 1.526, *p* = .002) on DAL and AE, respectively, were significant. However, the effects of HUM, EXP, and DEF on AE and DAL were nonsignificant. Lastly, the effects of AE on DAL were significant (b^DAL^ = 0.830, *p* = .000), which indicates that students who were more engaged were more likely to use DAL.

**Table 3:** Factor loadings of the structural equation model

Path			Unstandardized coefficient(B)	SE	Standardized coefficients($\mathbf{b}$)	SE
Growth mindset	→	CAR	0.523***	0.133	0.626***	0.133
Fixed mindset	→	CAR	0.292*	0.133	0.336*	0.141
**Growth mindset**	→	**PER**	0.566***	**0.135**	0.677***	**0.134**
Fixed mindset	→	PER	0.019	0.118	0.022	0.136
**Growth mindset**	→	**HUM**	0.352**	**0.110**	0.514***	**0.131**
Fixed mindset	→	HUM	−0.043	0.088	−0.060	0.126
Growth mindset	→	EXP	−0.049	0.083	−0.081	0.133
**Fixed mindset**	→	**EXP**	0.276**	**0.097**	0.441**	**0.134**
Growth mindset	→	DEF	−0.061	0.127	−0.054	0.113
**Fixed mindset**	→	**DEF**	0.599***	**0.138**	0.476***	**0.104**
CAR	→	AE	0.399	0.357	0.082	0.084
EXP	→	AE	−7.969	5.234	−1.388	0.888
**PER**	→	**AE**	6.310**	**2.084**	1.526**	**0.498**
DEF	→	AE	4.563	2.970	1.492	0.965
HUM	→	AE	−2.696	1.682	−0.534	0.318
**CAR**	→	**DAL**	−0.160	0.046	−0.164*	**0.083**
EXP	→	DAL	−0.242	0.083	−0.178	0.449
PER	→	DAL	−0.030	0.605	−0.030	0.396
DEF	→	DAL	0.094	0.388	0.130	0.482
HUM	→	DAL	0.260	0.348	0.218	0.219
**AE**	→	**DAL**	0.197***	**0.265**	0.830***	**0.160**

*Note* CAR: careerist–materialist motive, PER: personal–intellectual development motive, HUM: humanitarian motive, EXP: expectation-driven motive, DEF: default motive, DAL: deep approach to learning. Significant results are marked in bold

*p < .05, **p < .01, ***p < .001

### Mediation effects

Table 4 reports the unstandardized, standardized, and significance levels of the mediation effects for the model. AE did not mediate the association between CAR and DAL, whereas the direct path from CAR to DAL was negatively significant. AE mediated the association between PER and the DAL (mediation effect = 1.267), whereas the direct path from PER to DAL was nonsignificant, which denotes that AE fully mediated the relationship between PER and DAL.

**Table 4:** Mediation effects of the structural equation model

Path			Unstandardized coefficient ($\mathbf{B}$)	SE	Standardized coefficients ($\mathbf{b}$)	SE
CAR	→ AE	→ DAL	0.067	0.067	0.068	0.068
**PER**	→ AE	→ DAL	1.240*	**0.529**	1.267*	**0.526**
HUM	→ AE	→ DAL	−0.530	0.364	−0.444	0.295
EXP	→ AE	→ DAL	−1.567	1.142	−1.153	0.810
DEF	→ AE	→ DAL	0.897	0.643	1.239	0.158

*Note* CAR: careerist–materialist motive, PER: personal–intellectual development motive, HUM: humanitarian motive, EXP: expectation-driven motive, DEF: default motive, DAL: deep approach to learning. Significant results are marked in bold

*p < .05

## Discussions

This study aimed to examine the relationship among the mindsets, MAU, AE, and DAL of dental students and to test the mediating effect of AE on the relationship between MAU and DAL. The results partially confirmed the hypothesis model. In the structural equation model, the growth mindset was associated with CAR, PER, and HUM among the subfactors of MAU, whereas the fixed mindset was related to CAR, EXP, and DEF. Among the relationships between the subfactors of MAU and DAL, AE fully mediated that between PER and DAL. In addition, CAR directly and negatively predicted DAL without mediation. These findings suggested that the reasons for entering dental schools reflect the mindset of students about their growth in intellectual abilities through effort and learning. The results demonstrate that one’s belief in the potential for intellectual growth, dedication to self-improvement, and mastery of challenging tasks are significantly related to personal growth and the desire to help others. Conversely, a fixed mindset and the avoidance of challenging tasks due to perceived unalterable defects are associated with motives related to the search for approval from others and the absence of any clear motives.

Notably, among the subfactors of MAU, only CAR demonstrates a significant relationship with growth and fixed mindsets. A potential explanation is that CAR represents a complex nature. In other words, students who predominantly possess this motive may exhibit a blended mindset and intend to pursue intrinsic (e.g., fulfilling career and professional development) and extrinsic (e.g., wealth and prestige) goals [48]. This blended characteristic is likely adaptable and flexible, because it considers one’s internal aspirations and external constraints, which can influence future outcomes and opportunities [9]. 

Despite the characteristics of MAU as explained by the mindsets, the mediation model in this study illustrates that AE fully mediated only between PER and DAL. Corroborating previous findings on the positive relationships among intrinsic motivation, AE, and DAL [49–52], the current findings indicate that students attending dental school for personal growth and intellectual development can be academically more immersed than do those with other types of motives.

Initially, we hypothesized that a positive relationship exists between HUM and AE as well as DAL. However, after controlling for the statistical influence of PER in the proposed model, the predictive power of HUM became nonsignificant, which indicates that PER may exert a greater influence on these variables than does HUM. Although this study cannot directly confirm the underlying cause of this outcome, we propose a potential explanation. In the context of dental education, personal growth and intellectual advancement may be linked to the search for meaning in learning, while the desire to help others or promote community development may emerge from personal values. When interpreting these findings through the lens of self-determination theory [53], PER may be more relevant to intrinsic self-regulation (centering on activities and deriving positive affect from them), while HUM may apply to identified and integrated regulation (sustaining engagement in tedious but valuable activities). [54] Moreover, previous studies pointed to differences in these types of regulation. For example, Burton et al. [54] demonstrated that high levels of intrinsic regulation led to psychological well-being through activity enjoyment, while elevated levels of identified regulation link to favorable outcomes due to sustained long-term goal pursuit. Additionally, Yeager et al. [55] found that intrinsic self-transcendent motives (e.g., helping others and improving the world) may increase persistence in tedious tasks. Therefore, PER, when related to the meaningfulness of academic pursuit, may better predict AE and DAL, which involve positive academic feelings, compared with HUM.

Despite these results, HUM cannot be overlooked in the dental curriculum. In terms of social responsibility, dental schools have highlighted importance of professionalism, such as empathy, compassion, and altruism, as one of the core competencies. Additionally, academic work in these fields requires enduring tedious tasks and combating against the temptation to quit or persist in tedious tasks. Therefore, apart from initial admission motives, consistently aiming to enhance HUM via education in the dental school curriculum is imperative.

Apart from the mediational relationship, the study found that CAR is directly negatively associated with DAL. Previous studies demonstrated the relationships between high levels of materialism and low levels of engagement and intrinsic mastery goal [56, 57]. Given that intrinsic motivation and engagement are prerequisite factors for DAL [26], the materialistic motives of students render them less likely to take DAL by dampening one’s enthusiasm and mastery orientation for learning.

This study confirmed that the motives of dental students for selecting and entering a dental school influence AE and learning behaviors. In other words, identifying personal motives for admission should be conducted to support the successful academic performance of students. Moreover, identifying the motives for attending dental schools for new students and to follow up and analyze the effect of such motives on the entire academic process in relation to dental education is important. In addition, follow up studies should be conducted on whether the motives for selecting dentistry at the time of admission are maintained or changed during the academic year. This aspect poses significant implications in terms of student selection and guidance.

Furthermore, this study underscores the importance of fostering a growth mindset in students who have extrinsic goals or lack clear objectives in their healthcare education, to enhance intrinsic motivation and engagement. Common growth mindset strategies include tracking mindset changes over time, teaching the growth versus fixed mindset concept, and promoting progress-focused learning goals [58]. Developing a growth mindset can boost intrinsic motivation, encourage learning engagement, and improve academic performance. However, interventions alone may be insufficient; thus, a supportive healthcare education environment is essential. According to the mindset-plus-supportive-context hypothesis [59], fostering interaction between instructors and students of growth mindset enhances the development and maintenance of mindset.

Research on the application of mindset interventions among health professional students is limited [60]. For example, a structured intervention for nursing students, which included instruction on mindset theories and neuroplasticity, viewing a video that illustrated how individuals can overcome failures to achieve success, and facilitating self-reflection on personal mindsets, and training in growth mindset-based study strategies, significantly improved their growth mindset and their application of effective study techniques [61]. However, studies focusing on growth mindset interventions are notably lacking in certain healthcare disciplines, particularly in dental education and among faculty members in healthcare schools. This deficiency highlights the imperative for more extensive research in these specific areas.

Taken together, it may be beneficial to suggest that dental schools implement comprehensive assessments covering student mindsets, motives for attending dental school, AE, and learning approaches at the outset of their academic careers. By integrating these assessments into the educational framework, students can gain deeper insights into their own and their peers’ academic behaviors. This integration facilitates the development of effective study strategies and provides faculty with a clearer understanding of student profiles. Additionally, involving faculty in these assessments could foster their self-awareness and stimulate discussions among colleagues aimed at fostering an educational environment that supports DAL.

This study has its limitations, which should be considered when reviewing the findings. First, the cross-sectional nature of the analyses prevents the assessment of causal relationships between the observed variables. Thus, future studies should longitudinally test the examined mediation hypothesis and replicate the findings in subsequent years of dental studies and across contexts. Furthermore, analyzing the detailed yearly tendencies of MAU among dental school students may provide additional insights into the findings of this study. Secondly, the objective of this research was to provide a comprehensive overview of dental school students as a whole, rather than to divide the findings by grade level. However, there is a possibility that the mindsets may manifest differently across each grade level. Consequently, further research is required to address the limitations of not being able to reflect the characteristics of each grade level. Another limitation is that the conclusions were based on only a single dental university, which imposes evident limits on the generalizability of the findings. In this regard, expanding the study subjects to represent the diverse characteristics of populations of dental students is necessary. In addition, identifying the common and unique MAU and learning behavior of dental students and comparing them with those of students in other medical schools or nonhealthcare schools will be meaningful.

## Conclusions

We investigated the relationship between mindsets, academic engagement, and learning approach concerning motives for attending dental school. It posits that a thorough understanding of students’ mindsets and motivations is essential. Such insight could enable both dental students and faculty to introspectively examine their personal attributes and educational experiences, thereby fostering a deep approach to learning and cultivating a more effective educational environment in dental education. Consequently, this research augments the existing literature on learning behaviors in dental students.

## Data Availability

The datasets of this article are available from the corresponding author on reasonable request.

## Acronym: Title; Definition

MAU: Motives for attending university; Motives for attending dental school in this study
PER: Personal–intellectual development motive; Interest in personal growth and intellectual development
HUM: Humanitarian motive; Helping others and improving the world
CAR: Careerist–materialist motive; Seeking a good career and high socioeconomic status
EXP: Expectation-driven motive; Meeting the expectations of family and friends
DEF: Default motive; Attending university without a clear reason
DAL: Deep approach to learning; Learning strategies that are driven by intrinsic motivation, including the pursuit of understanding, the seeking of meaning, and the integration of content with personal experiences
AE: Academic engagement; A positive academic experience characterized by students’ active participation, emotional dedication, and deep involvement in educational activities
